# Does chlorhexidine mouthwash, with an anti‐discoloration system, reduce tooth surface discoloration without losing its efficacy? A systematic review and meta‐analysis

**DOI:** 10.1111/idh.12402

**Published:** 2019-08-01

**Authors:** Bregje W. M. Van Swaaij, G. A. (Fridus) van der Weijden, Eric W. P. Bakker, Filippo Graziani, Dagmar E. Slot

**Affiliations:** ^1^ Department of Dental Hygiene, Hogeschool Arnhem Nijmegen University of Applied Sciences Nijmegen The Netherlands; ^2^ Department of Periodontology, Academic Centre for Dentistry Amsterdam (ACTA) University of Amsterdam, Vrije Universiteit Amsterdam Amsterdam The Netherlands; ^3^ Division Clinical Methods and Public Health, Academic Medical Centre (AMC) University of Amsterdam Amsterdam The Netherlands; ^4^ Department of Surgical, Medical Medical and Molecular Pathology and Critical Care Medicine University of Pisa Pisa Italy; ^5^ Department of Periodontology UCL Eastman Dental Institute London UK

**Keywords:** anti‐discoloration system, chlorhexidine, gingivitis, plaque, review, tooth surface discoloration

## Abstract

**Objectives:**

To investigate whether chlorhexidine mouthwash (CHX‐MW), with an anti‐discoloration system(ADS), is effective in preventing extrinsic tooth surface discoloration. Additionally, this paper seeks to evaluate whether CHX combined with an ADS maintains its efficacy with respect to reducing plaque and gingivitis scores.

**Material and methods:**

MEDLINE‐PubMed and Cochrane‐Central were searched up to October 2018 to identify eligible studies. Papers evaluating the effect of CHX‐MW+ADS compared to CHX without an ADS were included. A descriptive analysis and when feasible a meta‐analysis was performed.

**Results:**

Screening resulted in 13 eligible publications, presenting 16 comparisons. Six of these evaluated the MW in a non‐brushing model and ten as an adjunct to toothbrushing. A descriptive analysis demonstrated that the majority showed no differences in bleeding, gingivitis and plaque scores. This was confirmed by the meta‐analysis. In non‐brushing experiments, the difference‐of‐means (DiffM) for plaque scores was 0.10 (*P* = 0.45, 95%CI: [−0.15; 0.34]) and for the gingival index 0.04 (*P* = 0.15,95%CI: [−0.02; 0.11]). The DiffM in brushing studies for plaque scores was 0.01 (*P* = 0.29, 95%CI: [−0.01; 0.02]) and for the gingival index 0.00 (*P* = 0.87,95%CI: [−0.05; 0.06]). With respect to staining scores, the meta‐analysis revealed that in non‐brushing studies, the standardized mean difference was 3.19 (*P* = 0.0005,95%CI: [−3.98; −1.41]) while in brushing studies, the DiffM was 0.12 (*P* = 0.95,95%CI: [−3.32; 3.55]).

**Conclusion:**

There is moderate quality evidence from non‐brushing studies that the addition of an ADS to CHX‐MW reduces tooth surface discoloration and does not appear to affect its properties with respect to gingival inflammation and plaque scores. In brushing studies, there is also moderate quality evidence that ADS does not affect the anti‐plaque and anti‐gingivitis efficacy of CHX. The majority of comparisons and the meta‐analysis including these indicate no significant effect of ADS on tooth staining in situations where the mouthwash is used in addition to toothbrushing.

## 1 INTRODUCTION

Gingivitis and periodontitis are perhaps the diseases most common among humans.^1^ It has been established that teeth consistently surrounded by inflamed gingiva have a significantly higher risk of being lost than teeth surrounded by no or only slight inflammation. Persistent gingivitis represents a risk factor for periodontal attachment loss and tooth loss. These may have a negative impact upon speech, nutrition, quality of life and self‐esteem, and have systemic inflammatory consequences.^2^, ^3^

Gingivitis occurs due to the accumulation of an undisturbed layer of microbial plaque around the oral cavity and tooth surfaces.^4^ Dental plaque deposit, the primary aetiologic factor for gingival inflammation, can be prevented by attaining and maintaining high standards of daily plaque removal. A manual or power toothbrush is recommended as a primary means of reducing plaque.^2^ In addition, daily use of interdental cleaning devices ensures less interdental bleeding.^5^ Using these techniques is generally sufficient to obtain satisfactory oral health. In this way, periodontitis is preventable and leads to reduced rates of tooth loss and improved quality of life.^2^, ^6^

Nonetheless, effective patient self‐care is not an easy task for everyone. Many people fail to achieve optimal levels of oral care when just brushing their teeth with a dentifrice. If such mechanical cleaning is insufficient, chemical plaque control with adjunctive anti‐microbial agents can be considered.^2^, ^7^

The anti‐microbial agent most frequently advised is chlorhexidine mouthwash (CHX‐MW), which can be used as an adjunct to daily oral hygiene for the prevention or treatment of gingival inflammation. Furthermore, CHX‐MW can be prescribed after scaling and root planning or tooth extraction.^8^ In periodontal surgery, CHX can be prescribed as an temporary alternative to mechanical plaque control.^9^, ^10^, ^11^ A large body of literature exists that demonstrates the effectiveness of CHX‐MW. Systematic reviews show that in particular, the parameters of plaque reduction and gingivitis significantly improved for those using a CHX‐MW compared to those using a placebo.^12^, ^13^

Although CHX‐MW is currently the most effective anti‐microbial agent for reducing plaque and gingivitis, it does have several side effects. An increased calculus formation and decreased taste sensation (hypogeusia) are often reported. Hypogeusia induced by CHX concerns specifically salt and bitter. Salt perception will reach the lowest value on the second day of treatment while the bitter perception on the seventh day, in general, does not change till mouthrinses were interrupted.^14^ Other less frequent complaints are a burning sensation, hypersensitivity, mucosal lesions and anaesthetized sensation.^12^ However, its major side effect is extrinsic tooth staining, which may have a negative effect on patient compliance with rinsing.^9^, ^12^, ^14^

For more than a decade, several commercial CHX‐mouthwashes with an anti‐discoloration system (ADS) have been available in different countries. Several studies have been performed; however, the results published regarding its effectiveness have been inconclusive.^15^, ^16^ It has been observed that an ADS can be effective in reducing stain, but it may potentially also reduce the clinical efficacy of CHX products.^16^ This has been summarized in the past in the following simplified manner: "if it does not stain, it does not work.”^17^

The purpose of this systematic review (SR) is to synthesize the available scientific literature to investigate whether adding an ADS to CHX‐MW is effective in preventing extrinsic tooth surface discoloration, as well as evaluating whether CHX combined with an ADS maintains its efficacy with respect to reduction of plaque and gingivitis.

## 2 MATERIAL AND METHODS

The preparation and presentation of this SR is in accordance with the *Cochrane Handbook for Systematic Reviews of Interventions* 18 and the guidelines of Transparent Reporting of Systematic Reviews and Meta‐Analyses (PRISMA).^19^ A protocol^20^ was developed a priori following the initial discussion between the members of the research team. The focused questions of the review were as follows:

- The first focused question: What is the effect of rinsing with a CHX‐MW containing an ADS, as opposed to rinsing with a standard CHX‐MW, on tooth surface discoloration?
- The second focused question: What is the effect of rinsing with a CHX‐MW containing an ADS, as opposed to rinsing with a standard CHX‐MW, on plaque and gingivitis scores?

### 2.1 Search strategy

A structured search strategy was designed to retrieve all relevant studies that evaluated the effectiveness of CHX‐MW, with and without an ADS, on the parameters of surface discoloration, plaque and gingivitis. The searches were independently executed by two reviewers (BVS and DES). The National Library of Medicine, Washington D. C. (MEDLINE‐PubMed) and the Cochrane Central Register of Controlled Trials (CENTRAL) were searched from the inception of this study to February 2019 for appropriate papers that answered the focused questions. The reference lists of the studies included in this meta‐analysis were hand searched to identify additional potentially relevant studies. Furthermore, the following database sources were searched for possible relevant studies that were either unpublished or published in non‐commercial form: OpenGrey (http://opengrey.eu/), the European Federation of Periodontology (http://efp.org) and the International Association for Dental Research (http://www.iadr.org). CHX product companies involved in the field of ADSs were contacted in an effort to trace unpublished or ongoing studies. Table 1 provides details regarding the search terms used. There were no restrictions regarding language or publication year.

**Table 1:** Search terms used for Pub Med‐MEDLINE and Cochrane‐CENTRAL. The search strategy was customized according to the database being searched. The following strategy was used in the search: {[<ingredient: CHX>] AND [<carrier: mouthwash>] AND [<addition: ADS>]}

{ [<ingredient: CHX>] [("Chlorhexidine"[Mesh]) OR chlorhexidine OR (chlorhexidine di‐gluconate) OR (chlorhexidine gluconate) OR (zinc‐chlorhexidine) OR (chlorhexidine glucona te lidocaine hydrochloride) OR CHX OR (CHX formulations) OR (chlorhexidine phosphanilate) OR (chlorhexidine di‐acetate)] AND [<carrier: mouthwash>] ["Mouthwashes"[Mesh]) OR (Mouthwashes OR Mouthwash OR mouthwash* OR mouthrinses OR mouthrinse] AND [<addition: ADS>] [(Anti‐discoloration system) OR ADS OR (anti‐discoloration system) OR Curasept] }

Note

The asterisk (*) was used as a truncation symbol.

### 2.2 Screening and selection

Initially, the titles and abstracts (when available) of all studies identified through the searches were scanned by two reviewers independently (BVS and DES), who then selected studies that potentially met the inclusion criteria. After this phase, full‐text versions were obtained for the studies that appeared to meet the inclusion criteria or for which the title and abstract provided insufficient information to make a clear decision. These studies were then categorized as “definitely eligible,” “definitely not eligible” or “questionable.” Disagreements concerning eligibility were resolved by consensus or, if disagreement persisted, by arbitration through a third reviewer (GAW). The papers that fulfilled all inclusion criteria were processed for data extraction. No language restriction was imposed.

The inclusion criteria were as follows:

- Randomized controlled trials (RCTs) or controlled clinical trials (CCTs)
- Trials conducted in humans participants who:
  o Are in satisfactory general health (no systemic disorder)
  o Are aged ≥18 years
  o Do not have partial or complete dentures
  o Do not have fixed orthodontic equipment
  o Do not have dental implants
  o Are not undergoing periodontal (flap) surgery
- Intervention: CHX‐MW+ADS
- Comparison: CHX‐MW
- Identical CHX concentration in intervention and control groups as first choice, only if this is not available, this is omitted.
- Rinsing regimen:
  o Daily rinsing with a minimum of twice daily CHX use
- Outcome parameters relevant to the focused questions:
  o First question: discoloration
  o Second question: plaque, bleeding or gingivitis scores

### 2.3 Methodological quality assessment

Two reviewers (BVS and DES) independently scored the individual methodological qualities of the studies included in this meta‐analysis using the checklist presented in Appendix S1. Quality criteria were designated with a positive sign (+) if an informative description was present, and if the study design met the methodological criteria, a negative sign (‐) if an informative description was present but the study design did not meet the criteria and a question mark (?) if information was missing or insufficient. A study was classified as having a “low risk of bias” when positive scores (+) were assigned to the criteria of random allocation, defined inclusion/exclusion criteria, blinding to product and examiner, balanced experimental groups, identical treatment between groups (except for the intervention) and reporting of follow‐up. Studies that had six of these seven criteria were considered to have a potential “moderate risk of bias”. If two or more of these seven criteria were absent, the study was considered to have a “high risk of bias”.^21^

### 2.4 Data extraction

All studies that met the inclusion criteria were selected for data extraction and a “risk of bias” assessment. Independent data extraction was performed by two reviewers (BVS and DES) using a specially designed standardized data extraction form. Data recorded from the studies included here were based directly on the focus of the research questions, including details of the population, intervention, comparison outcome and study characteristics. Disagreement between the reviewers was resolved through discussion until a consensus was reached. Any persisting disagreements were resolved by discussion with a third reviewer (GAW). If any missing data or information were identified, an attempt was made to contact the authors of the publication to request additional information.

### 2.5 Data synthesis

#### 2.5.1 Assessment of clinical and methodological heterogeneity

The factors used to assess the clinical heterogeneity of the outcomes of the various studies were as follows: characteristics of participants, groups, variation of the CHX concentration in the MW, evaluation period, side effects and industry funding. Factors to assess the methodological heterogeneity were diversity in study design. When clinical or methodological heterogeneity was considered to be too high across studies, sources of heterogeneity were investigated with subgroup and/or sensitivity analyses. When the individual studies were sufficiently similar with respect to included patients, treatments and outcomes, pooling of results was considered and statistical heterogeneity assessed.

2.5.2

##### 2.5.2.1 Assessment of statistical heterogeneity

Poor overlap of confidence intervals generally indicates the presence of statistical heterogeneity. Heterogeneity was statistically tested by the chi‐square and *I* ^2^ tests. Tau‐squared was used to estimate the between‐studies variation. A chi‐square test resulting in a *P* < 0.1 is considered an indication of significant statistical heterogeneity. As an approximate guide to assessing the possible magnitude of inconsistency across studies, an *I* ^2^ statistic of 0‐40% was interpreted to indicate unimportant levels of heterogeneity. An *I* ^2^ statistic of 30%‐60% may represent moderate heterogeneity an *I* ^2^ statistic of 50%‐90% may represent substantial heterogeneity while a statistic of greater than 75% was interpreted to indicate considerable heterogeneity. This form of heterogeneity was assessed with subgroup and or sensitivity analysis to assess the effect modification.^22^

#### 2.5.3 Descriptive methods

As a summary of data, a descriptive data presentation was used for all studies. It was decided “a priori” to categorize the studies into either monotherapy studies (non‐brushing studies) or studies that also included self‐performed daily oral hygiene (brushing studies). Discoloration scores, plaque, bleeding and gingivitis were taken into account.

#### 2.5.4 Quantitative methods

If quantitative methods were feasible, a meta‐analysis was performed to explore the effectiveness of CHX‐MW+ADS vs CHX‐MW alone within various parameters. Analysis was carried out using Review Manager version 5.3 according to the PRISMA guidelines.^19^ In studies consisting of multiple treatment arms, and in which data from one particular group were compared to the data of more than one other group, the number of subjects (n) in the group was divided by the number of comparisons. In cases where it was not possible to perform a meta‐analysis, only a descriptive analysis is reported. A meta‐analysis was performed if more than one study could be included.

When the pooled outcome of several studies was measured using the same unit, then it was expressed as a difference‐of‐means (DiffM) with its associated 95% confidence interval. When the primary outcome was measured using different units across studies, then the standardized mean difference (SMD) was used to combine the outcomes in the meta‐analyses.^23^

The DiffM between test and control was calculated using both the “random and fixed effects” model where appropriate. When there is heterogeneity that cannot readily be explained, one analytical approach was incorporated into a random‐effects model. Random‐effects models are well suited for meta‐analysis with heterogeneous effects. A fixed‐effect model was presented if there were fewer than four comparisons, because the estimate of between‐study variance is poor for analyses with low numbers of studies.^18^

The testing for publication bias per outcome was used as proposed by Egger et al.^24^ If the meta‐analysis involved sufficient trials to make visual inspection of the plot meaningful (a minimum of 10 trials), funnel plots were used as a tool to assess publication bias. The presence of asymmetry in the inverted funnel would suggest a systematic difference between large and small trials in their estimates of treatment effects—a difference that may occur, for example, because of publication bias.^18^, ^19^

#### 2.5.5 Grading the “body of evidence”

The Grading of Recommendations Assessment, Development and Evaluation (GRADE) was used to rank the evidence.^25^, ^26^ Two reviewers (BVS and DES) rated the quality of the evidence and the strength and direction of the recommendations^27^ according to the following aspects: risk of bias, consistency of results, directness of evidence, precision, publication bias and magnitude of the effect. Any disagreement between the two reviewers was resolved through additional discussion.

## 3 RESULTS

### 3.1 Search and selection results

**Figure 1:**
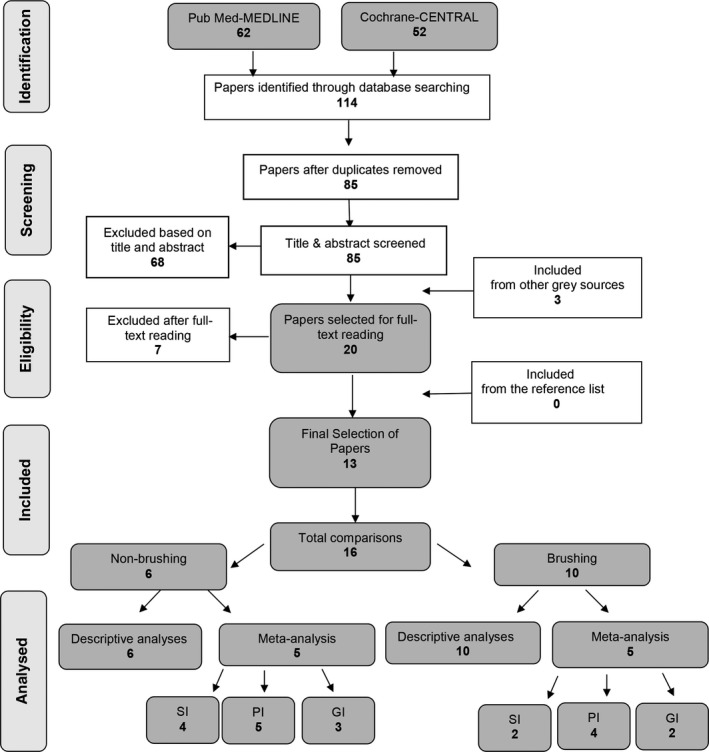
Search and selection results. SI=staining index, PI=plaque index , GI= gingival index

Searching the MEDLINE‐PubMed and Cochrane‐CENTRAL databases resulted in 85 unique papers (Figure 1). Screening of the titles and abstracts narrowed the results to 20 papers for which the full reports were obtained. Based on a detailed reading of the full texts, 13 papers were selected. Manually searching the reference lists and contacting manufacturers did not result in additional publications. The 13 eligible papers provided 16 comparisons. Of the three papers that contributed with double comparisons, one study (X^28^) compared ADS to two different commercially available CHX‐MW brands. The other two studies (IV^29^ and IX^30^) both made a comparison between ADS and an alcohol‐containing or an alcohol‐free CHX‐MW.

### 3.2 Assessment of clinical heterogeneity

Heterogeneity was observed in the 13 clinical trials with respect to participants, and mouthwash (MW) brands used in the brushing/rinsing regimen among the studies. Table 2 presents information regarding the characteristics of the studies included in this meta‐analysis.

**Table 2:** Overview of the studies processed for data extraction

# Study Authors (year)	Study design, duration	Participants base(end), Gender, Age (mean/range), Oral prophylaxis (OP)	Groups *Brands* Regimen	Conclusions of the original authors
I^15^ Bernardi et al (2004) Brushing	RCT Crossover Single‐blind 14 d	15 (15) ♀: ? ♂: ? Mean age: ? Age range: 20‐60 OP: Yes	CHX‐MW 0.2% + ADS, *Curasept, Curaden* *Healthcare; Saronno, IT* CHX‐MW 0.2% alc? 12 mL for 60 s twice a day	There is no statistically significant difference in the ability of the CHX‐MW to prevent bacterial plaque,; however, evidence of the stain was much less with the CHX‐MW + ADS
II^36^ Arweiler et al (2006) Non‐brushing	RCT Crossover Single‐blind 4‐day plaque regrowth study	21 (19) ♀: 13 ♂: 6 Mean age: 29.1 Age range: 20‐52 OP: Yes	CHX‐MW 0.2% + ADS, *Curasept* *ADS 220, Curaden* *AG; CH* CHX‐MW 0.2% *Chlorhexamed* *forte (Corsodyl), GSK; DE* alc + 10 mL for 60 s twice a day	The results of this study suggested that the 0.2% alcohol‐containing solution showed superiority in inhibiting plaque re‐growth and reducing bacterial vitality compared with the solution with ADS
III^37^ Basso et al (2005 + 2008) *(Two papers reporting the same experiment)* Non‐brushing	Cohort Crossover Double‐blind 15 d	30 (30) ♀: ? ♂: ? Mean age: Age range: 19‐39 OP: Yes	CHX‐MW 0.2% + ADS, *Curasept, Curaden* *Healthcare; Saronno, IT* CHX‐MW 0.2% *(Brand unknown)* alc‐ 15 mL for 60 s twice a day	CHX‐MW + ADS appears useful in tooth staining reduction, without being less effective in clinical application
IV^29^ Graziani et al (2009) Brushing	RCT Parallel Double‐blind 30 d	40(40) ♀: ? ♂: ? Mean age: ? Age range: ? OP: Yes	CHX‐MW 0.2% + ADS *Curasept, Curaden* *Healthcare SpA Saronno* *VA Italy* CHX‐MW 0.2% *Corsodyl, GSK; Milan, IT* alc + CHX‐MW 0.2% *Dentosan, J&J; Rome, IT* alc‐ ? ml for 60 sec twice a day	It appears that the mouthrinse which produces discoloration is the most effective in plaque control
V^31^ Solis et al (2010) Brushing	RCT Crossover Double‐blind 15 d	17 (15) ♀: 7 ♂: 8 Mean age: 55.47 Age range: 35‐69 OP: Yes	CHX‐MW 0.2% + ADS *Curasept, Curaden* *Healthcare, Curadent* *Int. AG; Kriens, CH* CHX‐MW 0.2% (Brand unknown) alc? 10 mL for 60 s twice a day	The CHX‐MW + ADS had less staining than the CHX‐MW group during a usage period of 15 d. The two mouthwashes seemed to be equally effective as anti‐plaque and anti‐gingivitis agents
VI^32^ Amato et al (2012) Brushing	RCT Parallel 14 d After scaling/planing	30 (30) ♀: 13 ♂: 17 Mean age: 48 Age range: ? OP: Yes	CHX‐MW + NST 0,2% PlakOut Active alc‐CHX‐MW 0,2% *Brand unknown* alc‐ 10 mL for 60 s twice a day	Treatment for 2 wk with alcohol‐free 0.2 chlorhexidine mouthwash containing an anti‐discoloration system allows good control of mucobacterial plaque without causing tooth discoloration
VII^16^ Li et al (2013) Non‐brushing	RCCT Parallel Double‐blind 21 d Experimental gingivitis model	26 (26) ♀: ? ♂: ? Mean age: ? Age range: 18‐20 OP: Yes	CHX‐MW 0.12% + ADS *Curasept* *ADS 212,* CHX‐MW 0.12% *(Brand of origin unknown)* alc? 10 mL for 60 s twice a day	The CHX‐MW + ADS appeared to be effective in preventing stain on teeth; however, it did not prevent plaque or gingivitis development. The CHX‐MW + ADS showed no superior effect over placebo on the prevention of gingivitis
VIII^38^ Weinstein & Weinstein (2014) Non‐brushing	RCT Crossover Triple‐blind 14 d	50 (50) ♀: ? ♂: ? Mean age: ? Age range 18‐? OP: Yes	CHX‐MW 0.09% + ADS + 0.2% PVP‐VA *Curasept* *(place unknown)* alc‐ CHX‐MW 0.2% *Chlorhexamed* *forte* *(place of origin unknown)* alc‐ 10 mL for 60 s twice a day	PVPA 0.2% mouthrinse demonstrates a similar activity in respect to a CHX 0.2% in inhibiting plaque and preventing gingivitis. PVPA 0.2% mouthrinse with ADS leads to lower formation of tooth staining in respect to a conventional CHX 0.2% mouthrinse
IX^30^ Graziani et al (2015) Brushing	RCCT Parallel Double‐blind 35 d IDC	55◊ (55◊) ♀: 28◊ ♂: 27◊ Mean age: 35.6◊ Age range: ? OP: Yes	CHX‐MW 0.2% + ADS *Curasept, Curaden* *Healthcare; Saronno, IT* CHX‐MW 0.2% *Dentosan, J&J; Rome, IT* alc‐ CHX‐MW 0.2% *Corsodyl, GSK; Milan, IT* alc+ 10 mL for 60 s twice a day	Conventional CHX‐MW appeared more effective in terms of plaque reduction. The CHX‐MW + ADS formulation showed a higher control of gingival inflammation. Staining was associated with lower plaque levels
X^28^ Marrelli et al (2015) Non‐brushing	RCT Parallel Double‐blind 15 d	200 (200) ♀: 128◊ ♂: 72◊ Mean age: ? Age range: 18 to <50 OP: Yes	CHX‐MW 0.2% + ADS *Curasept, Curaden* *Healthcare; Saronno, IT* CHX‐MW I 0.2% *Curasept, Curaden* *Healthcare; Saronno, IT* CHX‐MW II 0.2% *(Brand unknown)* alc? 10 mL for 60 s twice a day	CHX + ADS is clinically effective in the reduction of tooth staining without a loss of anti‐plaque activity, with respect to the competing mouthwashes containing CHX
XI^33^ Pereira et al (2017) Brushing	Cohort Crossover Triple‐blind 15 d	15 (15) ♀: ? ♂: ? Mean age: ? Age range: 18‐25 OP: Yes	CHX‐MW + ADS, 1% Plasdone‐ molecule *HEXIDINE‐EP* alc‐ CHX‐MW 0.2% *Brand unknown* alc? 10 mL for 60 s twice a day	The addition of Plasdone does not reduce the efficacy of CHX‐MW. The current study also proved that CHX‐MW + ADS caused less staining than CHX alone
XII^34^ Varoni et al (2017) Brushing	RCT Crossover Wash out 21 d	22 (22) ♀: 8 ♂: 14 Mean age: 25.5 Age range: 18‐40 OP: Yes	CHX‐MW + ADS 0.12% *Curasept, Curaden* *Healthcare; Saronno, IT* CHX‐MW 0.12% *Dentosan, J&J, Milan, IT* alc? 20 mL for 60 s twice a day	A slight discoloration was the most frequent finding, independent of the presence of ADS, while the few severe cases of staining were associated with CHX alone. Direct visual analysis showed no staining difference between the two mouthwashes
XIII^35^ Guerra et al (2019) Brushing	RCT Parallel Double‐blind 14 d	66 (64) ♀: 40 ♂: 26 Mean age: 29.3 Age range: 18‐40 OP: Yes	CHX‐MW 0.2% + ADS *Curasept, Curaden* *Healthcare SpA Saronno* *VA Italy* CHX‐MW 0.2% *Dentosan recordati SpA* *Milan, Italy* alc‐ 10 mL for 60 s twice a day	ADS addition decreases CHX effectiveness in reducing plaque and bleeding, while resulting more tolerated than CHX alone

Note

OP = At initial appointment, all teeth were thoroughly scaled and polished; PVP‐VA = Polyvinylpyrrolidon Vinylacetat; ? = unknown/not provided; ◊ = calculated by the authors of this review based on the presented data in the selected paper.

Abbreviations: IDP, Interdental Brushes; MW, Mouthwash; TP, Toothpaste.

Eight studies (I^15^, IV^29^, V^31^, VI^32^, IX^30^, XI^33^, XII^34^ and XIII^35^) used the MW as an adjunct to self‐performed daily oral hygiene. Study duration ranged from 14 to 35 days. The other five studies were non‐brushing studies with rinsing durations of 4 to 21 days(II^36^, III^37^, VII^16^, VIII^38^ and X^28^).

The concentration of CHX in the MW products was, in the majority of the studies, 0.20%. A concentration of 0.12% was used in Studies VII^16^ and XII^34^, and a concentration of 0.09% was used in Study VIII^38^. The concentration of CHX in the comparison products was similar in every study except for study VIII^38^, that is, 0.09% in the CHX‐MW+ADS compared to 0.20% in the CHX‐MW. Whether a given CHX‐MW contained alcohol was frequently not mentioned. Every study except two (XI^33^ and VI^32^) instructed the use of the CHX‐MW+ADS, which did not contain alcohol. For comparison, several brands were used. The rinsing regimen was set at twice daily for 60 seconds each. Different volumes of rinsing solutions were used from 10 mL up to 20 mL; only Study IV^29^ did not specify the volume.

The populations under evaluation in Studies VII^16^ and XI^33^ were dental students, and Studies II^36^ and XII^34^ included dental care professionals (dental students, dentists and dental hygienists). For inclusion in the individual studies, the following definitions, criteria and diagnoses were used regarding oral hygiene and periodontal health: no gingivitis (I^15^) no pockets ≥4 mm that bleed upon probing (VII^16^) and no periodontitis (XII^34^). Study XIII^35^ included patients presenting with a gingival index between 1.1 and 2.0. Periodontitis patients were specifically included in two studies (IV^29^ and VI^32^). Other criteria used in various studies included the following: a plaque index of <1 (XI^33^), and a Papilla Bleeding Index of <40% (II^36^).

Diet restrictions are described in the majority of the included studies. In four studies, the participants were instructed to refrain from tea, coffee and red wine intake for at least 1 hour before/after rinsing (I^15^, III^37^, V^31^ and IX^30^). In study X^28^, participants were advised to limit the chewing and drinking of chromogenic foods such as tea, coffee, red wine and spinach. Study II^36^ did not allow chewing gum. Participants of study XI^33^ were asked not to eat or drink for 30 minutes after rinsing. In study XII^34^, diet was recorded at baseline, and for the entire period, the participants were asked to maintain their usual lifestyle. Study XIII^35^ has excluded patients who take more than two cups of tea/coffee/red wine daily and usually eat liquorice. In four studies, no diet restrictions were reported (IV^29^, VI^32^, VII^16^ and VIII^38^).

#### 3.2.1 Side effects

The papers used in this meta‐analysis did not report any serious adverse effects. Most studies mentioned staining as a side effect of CHX during the experiments. The CHX‐MW+ADS group in Study XII^34^ reported less taste alteration, while in Study VIII^38^, this was reported for the CHX‐MW group.

#### 3.2.2 Industry funding

Three studies do not mention any details regarding funding or conflict of interests (I^15^, II^36^ and III^37^). Seven studies specifically mentioned not having a conflict of (financial) interest (V^31^, VI^32^, VII^16^, VIII^38^, X^28^, XII^34^ and XIII^35^). Some studies mentioned a relation with industry. Study samples with CHX‐MW were provided by Curaden (IV^29^ and V^31^), ICPA Pharmaceuticals (XI^33^), GlaxoSmithCline (IV^29^) and Johnson and Johnson (IV^29^ and XII^34^). Funding was declared by related industries such as Johnson and Johnson (IX^30^ and XII^34^) and Curaden (V^31^). Study X^28^ mentioned that employees from Curaden had contributed to the study design and the analysis of the study. A sensitivity analysis on funding or industry relation was not possible.

### 3.3 Assessment of methodological heterogeneity

All studies were RCTs, of which seven used a crossover design (I^15^, II^36^, III^37^, V^31^, VIII^38^, XI^33^ and XII^34^) and six used a parallel design (IV^29^, VI^32^, IX^30^, VII^16^, X^28^ and XIII^35^).

### 3.4 Methodological quality assessment

The potential risk of bias was estimated based on the methodological quality aspects of the selected studies, as presented in the online Appendix S1. Based on a summary of the proposed bias‐assessment criteria, the potential risk of bias was estimated to be moderate for Studies I^15^ and III^37^ and low for the other studies. Sub‐analysis was performed only for studies with a low risk of bias.

### 3.5 Study outcome results

The online Appendix S2, sub‐sections a–d, presents the results of the data extraction that was performed on the selected studies in various clinical indices. When available, the baseline, end scores and changes between baseline and end scores are presented.

#### 3.5.1 Description of findings

In detail, Table 3 described and summarizes the statistical differences between CHX‐MW+ADS and CHX‐MW, presented for the brushing and non‐brushing studies.

**Table 3:**
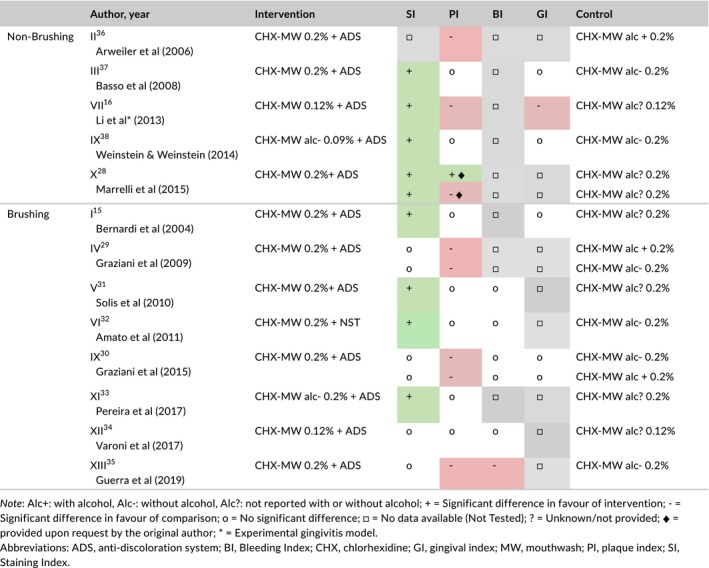
A descriptive summary of statistical significance levels of the use of chlorhexidine mouthwashes with or without an antidiscoloration system, with or without alcohol and without brushing or as adjuvant to toothbrushing on the parameters of interest

Note

Alc+: with alcohol, Alc‐: without alcohol, Alc?: not reported with or without alcohol; + = Significant difference in favour of intervention; ‐ = Significant difference in favour of comparison; o = No significant difference; □ = No data available (Not Tested); ? = Unknown/not provided; ♦ = provided upon request by the original author; * = Experimental gingivitis model.

Abbreviations: ADS, anti‐discoloration system; BI, Bleeding Index; CHX, chlorhexidine; GI, gingival index; MW, mouthwash; PI, plaque index; SI, Staining Index.

The majority of the 16 comparisons showed a statistically significant benefit in favour of CHX‐MW+ADS for a reduction in *stain* scores. In all but two comparisons (VII^16^ and XIII^35^), no statistical differences on the parameters of bleeding and gingivitis were obtained when an ADS was added. Plaque scores reveal an inconsistent pattern: seven comparisons showed no difference, seven showed that an ADS negatively influenced plaque score, and one positively influenced it.

#### 3.5.2 Meta‐analysis

It was possible to perform a meta‐analysis for the comparisons between products assessing stain scores for non‐brushing and brushing studies. For non‐brushing studies, a significant difference was found in the SMD for end scores as well as for the incremental difference (SMD = −3.19, *P* = 0.0005; 95% CI: [−3.98; −1.41] and SMD = −3.03, *P* = 0.0006; 95% CI: [−4.76; −1.30], respectively). When a study design that included toothbrushing was used, no significant differences were found between CHX‐MW+ADS and CHX‐MW. The DiffM for end scores of staining was 0.12 (*P* = 0.95; 95% CI: [−3.32; 3.55]). The treatment effect is assessed with the Silness & Löe plaque index^39^ for non‐brushing and brushing studies. In non‐brushing studies, no significant difference was found between the baselines of two groups. In addition, neither the DiffM of end scores (DiffM 0.10, *P* = 0.45; 95% CI: [−0.15; 0.34]) nor the incremental difference (DiffM 0.10, *P* = 0.46; 95% CI: [−0.16; 0.35]) were significant. This was supported by the end scores of brushing studies (DiffM 0.01, *P* = 0.29; 95% CI: [−0.01; 0.02]).

With respect to the Löe & Silness gingival index,^40^ the DiffM for non‐brushing studies was not significant neither at the baseline nor at the end, with a DiffM of −0.01 (*P* = 0.62; 95% CI: [−0.04, 0.02]) and a DiffM of 0.04 (*P* = 0.15; 95% CI: [−0.02, 0.11]), respectively. The end scores of the brushing studies in which toothbrushing was used as an adjunct to the CHX‐MW products support the findings that there is no difference between CHX‐MW+ADS and CHX‐MW (DiffM 0.00, *P* = 0.87; 95%CI: [−0.05; 0.06]). Table 4a,b summarizes the detailed data of the outcomes of the meta‐analysis. Online Appendices S3‐S8 present the corresponding forest plots. A test for publication bias could not be performed because fewer than 10 studies were included in the meta‐analysis, which would result in insufficient statistical power.^18^, ^24^ Consequently, publication bias cannot be ruled out. Sub‐analysis of studies that possessed a low risk of bias did not reveal any significant discrepancies with the original analysis. The heterogeneity is exposed and stays unclarified (Appendix S9a‐c).

**Table 4:** Meta‐analysis for the baseline, end and incremental data evaluating the efficacy of ADS added to CHX‐MW on plaque and gingival inflammation in (a) non‐brushing studies and (b) brushing studies

Index	Measurement moment	Included studies	Model	DiffM	Test overall		Test for heterogeneity		For details see online Appendix
					95% CI	*P*‐value	*I* ^2^ value (%)	*P*‐value	
(a) Non‐brushing									
SI Lobene (1968)^51^	Base	Li et al (2013) Marelli et al (2015)^a^ Weinstein & Weinstein (2014)	Random (SMD)	−0.39	[−1.09; 0.31]	0.27	87	<0.00001	S3a
	End	Basso et al (2008) Li et al (2013) Marelli et al (2015)^a^ Weinstein & Weinstein (2014)	Random (SMD)	−3.19	[−4.98; −1.41]	0.0005	97	<0.00001	S3b
	Diff	Basso et al (2008) Li et al (2013) Marelli et al (2015)^a^ Weinstein & Weinstein (2014)	Random (SMD)	−3.03	[−4.76; −1.30]	0.0006	97	<0.00001	S3c
PI Silness and Löe (1964)^39^	Base	Arweiler et al (2006) Li et al (2013) Weinstein & Weinstein (2014) Marelli et al (2015)^a^	Random	0.01	[−0.06; 0.08]	0.84	86	0.01	S4a
	End	Arweiler et al (2006) Basso et al (2008) Li et al (2013) Weinstein & Weinstein (2014) Marelli et al (2015)^a^	Random	0.10	[−0.15; 0.34]	0.45	99	<0.00001	S4b
	Diff	Arweiler et al (2006) Basso et al (2008) Li et al (2013) Weinstein & Weinstein (2014) Marelli et al (2015)^a^	Random	0.10	[−0.16; 0.35]	0.46	98	<0.00001	S4c
GI Löe and Silness (1963)^40^	Base	Li et al (2013) Weinstein & Weinstein (2014)	Fixed	−0.01	[−0.04; 0.02]	0.62	0	0.90	S5a
	End	Basso et al (2008) Li et al (2013) Weinstein & Weinstein (2014)	Fixed	0.04	[−0.02; 0.11]	0.15	97	<0.00001	S5b
	Diff	Basso et al (2008) Li et al (2013) Weinstein & Weinstein (2014)	Fixed	0.05	[−0.00; 0.11]	0.06	98	<0.00001	S5c
(b) Brushing									
SI CIE (1971)^52^	End	Varoni et al (2017) Graziani et al (2015)^a^	Fixed	0.12	[−3.32; 3.55]	0.95	0	0.76	S6a
GI Löe and Silness (1963)^40^	Base	Graziani et al (2015)^a^	Fixed	0.11	[−0.28; 0.49]	0.59	0	0.61	S8a
	End	Graziani et al (2015)^a^ Solis et al (2010)	Fixed	0.00	[−0.05; 0.06]	0.87	0	0.38	S8b
PI Silness and Löe (1964)^39^	End	Bernardi et al (2005) Pereira et al (2017) Solis et al (2010) Varoni et al (2017)	Random	0.01	[−0.01; 0.02]	0.29	26	0.26	S7a

a Two comparisons arriving from this study.

#### 3.5.3 Sensitivity analysis

In the meta‐analysis of those studies that evaluated the intervention under non‐brushing circumstances, considerable heterogeneity was observed. For instance, the meta‐analyses for stain scores showed an *I* ^2 ^of 87%, 97% and 97% for baseline, end and incremental scores, respectively. A sensitivity analysis was performed to explore the source of heterogeneity which showed that without the outlying study X^28^, lower heterogeneity was present between the outcomes of the studies both at baseline (*I* ^2^ = 0%) and for incremental scores (*I* ^2^ = 69). For the end score, the *I* ^2^ remained high (94%) for which no obvious explanation was found. The studies included in the meta‐analysis do differ by study design, being either crossover or parallel. In the meta‐analysis on plaque scores, the sensitivity analysis was performed by study design. Meta‐analysis that only included those with a crossover design showed a decrease in *I* ^2^ for end scores of plaque from 99% to 89%, and for incremental scores from 94% to 70%. When only the parallel designs are taken into account, no evident explanation was found as well. The *I* ^2^ still remained high. For gingival scores, if the study VII^16^ with the smallest sample (N = 8) size is excluded, the *I* ^2^ for end scores and incremental scores decreased from 94% and 95%, respectively, to 0% for both. None of these sensitivity analyses did affect the overall result and conclusions.

### 3.6 Evidence profile

Table 5 presents a summary of the various criteria with which the quality of the evidence was rated and with which the strength and direction of recommendations were appraised according to Guyatt et al. 2008.^41^ The addition of an ADS to CHX‐MW is favourable with respect to reducing tooth surface discoloration, and it does not appear to affect the inhibition of plaque and gingivitis scores. Given the strength of the recommendation, there is a weak‐to‐moderate certainty that the addition of an ADS does not negatively influence the effect of CHX‐MW on plaque scores and gingival inflammation. Given that only in studies with a non‐brushing design, it significantly reduces tooth surface discoloration, the direction of the recommendation for situations where toothbrushing is not involved is moderately in favour of the use of CHX‐MW+ADS.

**Table 5:** Summary of findings table based on the quality and body of evidence on the estimated evidence profile (Guyatt et al)^41^ and appraisal of the strength of the recommendation regarding the efficacy of ADS added to CHX‐MW on the parameters of interest

Study design	Staining		Plaque		Gingivitis	
	Non‐brushing	Brushing	Non‐brushing	Brushing	Non‐brushing	Brushing
# experiments descriptive analysis (Table 3)	4	8	5	8	3	4
# experiments in Meta‐analysis (Table 4)	4	2	5	4	3	2
Risk of bias (Appendix [Link])	Low‐moderate	Low‐moderate	Low‐moderate	Low‐moderate	Low‐moderate	Low‐moderate
Consistency	Rather consistent	Rather consistent	Inconsistent	Rather consistent	Rather consistent	Consistent
Directness	Direct	Direct	Direct	Direct	Direct	Direct
Precision	Rather precise	Imprecise	Precise	Rather precise	Precise	Rather precise
Reporting bias	Possible	Possible	Possible	Possible	Possible	Possible
Magnitude of the effect (Tables 3 and 4)	Large	Ambiguous	No difference	No difference	No difference	No difference
Strength and direction of the recommendation	Moderate quality evidence in favour of	Very weak quality evidence for no difference	Weak quality evidence for no difference	Moderate quality evidence for no difference	Moderate quality evidence for no difference	Moderate quality evidence for no difference
Overall recommendation	If CHX‐mouthwashes are prescribed for gingivitis and plaque reduction, and there is interest to reduce staining, there is moderate evidence for non‐brushing situations to consider a product containing an anti‐discoloration system					

## 4 DISCUSSION

There is a strong body of evidence in support of CHX‐MW^12^, ^13^; however, one of the most prominent side effects is tooth staining. The occurrence of such staining could influence the compliance of the patient with respect to the regular and proper use of CHX. The initial question was whether adding an ADS provides a benefit or not. The present study aimed to evaluate the efficacy of a CHX‐MW with an ADS, compared to a CHX‐MW without an ADS, on tooth surface discoloration. In addition, this study questions whether CHX is still active with respect to the parameters of plaque and gingivitis when combined with an ADS. This SR demonstrated that the majority of the individual experiments presented a statistically significant benefit favouring CHX‐MW+ADS in terms of stain scores. The majority also found no differences in bleeding, gingivitis and plaque scores between CHX‐MW+ADS and CHX‐MW. The latter was confirmed by the meta‐analyses while with respect to staining, the meta‐analyses showed a significant effect for non‐brushing studies which was not substantiated for brushing studies.

### 4.1 Interpreting of staining analysis

In the non‐brushing studies using a non‐brushing model, all six comparisons in the descriptive analysis (Table 3), except Study II,^36^ significantly favoured CHX‐MW+ADS for stain scores. This was confirmed by the meta‐analysis, based on five comparisons. It should be taken into account that the trials comparing CHX‐MW+ADS to CHX‐MW for tooth staining included subjects diagnosed with both gingivitis and periodontitis. The meta‐analysis for stain scores was performed using the standardized mean difference as a summary statistic because studies measure the outcome in a variety of ways.^18^

A total of ten comparisons used the mouthwash as an adjunct to toothbrushing. In the descriptive analysis (Table 3), four comparisons showed a significant difference in favour of CHX‐MW+ADS. The other six comparisons, originating from four papers (IV^29^, IX^30^, XII^34^ and XIII^35^), did not find a significant difference between the groups. The three comparisons that were included in the meta‐analysis indicate a lack of difference between the groups. It should however be noted that the majority of the comparisons of the brushing studies could not be included in the meta‐analysis due to a lack of data, even though the authors were contacted for additional information. Nevertheless, based on the combined data from the descriptive analysis of non‐brushing and brushing designs and meta‐analysis of the non‐brushing designs, the addition of an ADS to CHX‐MW appears favourable with respect to preventing tooth surface discoloration. As a result, the first question can be answered: CHX‐MW+ADS has the potential to reduce the side effect of tooth staining.

### 4.2 Possible mechanisms

The staining side effect associated with CHX rinsing is attributed to three possible mechanisms: (a) the Maillard reaction, (b) the formation of pigmented metal sulphides and (c) reactions between polyphenols and tannin from food and drinks and chlorhexidine itself. The Maillard reaction occurs between sugars and proteins in the biofilm. This is a reaction catalysed both by CHX and a series of polymerization reactions. Consequently, the coloured pigments also known as “melanoidins” are formed. According to the manufacturer, one of the components of an ADS (a patented system) reacts with diketosamine. By removing the diketosamine, the formation of melanoidins can be prevented. Other mechanisms of discoloration relate to the protein denaturation by CHX. This leads to the formation of organic yellow‐brown ferric sulphides through the reaction between the combination of hydrogen and sulphur with an iron present in saliva. This reaction is inhibited by a component of the ADS, which reduces the level of iron.

### 4.3 Anti‐microbial activity

The second important question is whether an ADS compromises the anti‐plaque and consequently the bleeding effect of CHX. The overall findings of this SR conflict with the results of some the individual papers involved in the analysis. Four studies that significantly favour CHX‐MW over CHX‐MW+ADS in terms of plaque control (II^36^, IX^30^, VII^16^ and X^28^) are included in the meta‐analysis. The study by Arweiler et al (II^36^) is a 4‐day plaque re‐growth study and did not evaluate the primary outcome of this SR, that is, tooth surface discoloration scores. This study did, however, demonstrate that after a professional oral prophylaxis, CHX‐MW was more effective in inhibiting plaque regrowth than CHX‐MW+ADS. The weaker antibacterial and anti‐plaque activity in their study can in part be explained by the addition of an ADS to the MW‐solution: The ADS should reduce the staining potential of CHX, but apparently at the cost of reducing plaque control benefits. The two ADS molecules metabisulphite and ascorbic acid may compete with the CHX molecule and inhibit the adhesion of the positively charged CHX molecule to the tooth surface and other intra oral structures. It seems plausible that these components may interfere with CHX.^36^ This would accord with findings indicating that a reduction in the tendency to stain may also lead to a loss of plaque inhibition. It is also possible that in vivo, there is a continuous competition between anti‐plaque and anti‐staining processes.

Another uncertainty is the difference in outcomes of “in vitro*”* compared to “in vivo*”* research regarding it clinical relevance. In an early “in vitro*”* study, it was shown that no significant difference in staining existed between the ADS rinses and the positive control rinse.^17^ In addition, in a polyspecies biofilm model, the effect of CHX‐MW+ADS was evaluated, showing that all solutions containing CHX significantly reduced the number of microorganisms in biofilms. The CHX without an ADS proved most effective in reducing the total number of bacterial colonies. It was therefore proposed that regular CHX mouth rinses are best confined to short‐term therapeutic use and the addition of ADS solutions would be indicated for a long‐lasting prophylactic application.^42^ This conflicts with the results of the current review. A similar phenomenon was observed when a sodium lauryl sulphate (SLS) dentifrice was used in combination with a CHX‐MW. In vitro, SLS and CHX may act as antagonists.^43^ Based on a recent SR of clinical trials, the combined use of an SLS‐containing dentifrice and CHX‐MW is not contraindicated.^44^ Therefore, it may be concluded that CHX does not act similarly in vitro compared to “in vivo*.”*

### 4.4 Research models

From the 13 papers included in this analysis, eight evaluated the MW as an adjunct to brushing, and five were non‐brushing comparisons. Of the non‐brushing studies, Study VII^16^ specifically mentioned the use of an experimental gingivitis model by Löe et al^45^ This model is frequently used and allows for the evaluation of the effect of an anti‐microbial agent on plaque accumulation and parameters of gingivitis, for instance, an agent incorporated into a MW.^46^ Part of a pre‐experimental period for this specific model is a professional prophylaxis and optimal self‐performed plaque control to establish a healthy gingiva.^46^ All non‐brushing experiments provide such a prophylaxis, but only Study VII^16^ also concluded the pre‐experimental preparatory phase of optimal oral hygiene practices. In addition, in the past, it has been proposed that the period without mechanical plaque control should extent over at least 14 days.^46^ The non‐brushing experiments included in this review varied from 4 to 28 days. Recognizing the observation reported by Löe et al,^45^ it may be concluded that the non‐brushing period may not be less than 14 days. If the duration of an experiment is shorter than this 14‐day test period, it is appropriate only to evaluate changes in plaque scores and not to draw conclusions with respect to gingivitis. That is, only a statement about the anti‐plaque efficacy of the anti‐microbial agent can be made.^46^ This being proposed, from Study II,^36^ with a duration of 4 days, only the plaque scores were extracted. All studies were RCTs but differed by study design, as seven used a crossover and six used a parallel model (for details see Table 2). Study designs may influence the heterogeneity.^18^ The present review excluded surgical procedures as part of a study protocol of interest. The search revealed that some papers are published on the topic CHX with or without ADS as adjunct used by periodontal flap surgery.^10^, ^47^, ^48^ Non‐surgical periodontal therapy differs from resective or regenerative procedures by its origin and indication. As the non‐brushing studies in this review mostly refer to experimental gingivitis conditions, and not post‐surgery use of the mouthwash, it seems of interest to evaluate the staining properties with the specific study model of periodontal surgery in a future systematic review.

### 4.5 Clinical and methodological heterogeneity

Out of the 13 included studies, two (V^31^ and XI^33^) had industry involvement. It is well‐established that publication bias may be associated with the source of funding for a study. The main origin of this bias is failure to publish negative or null findings. The consequence is that it may lead to overestimates of treatment effects in meta‐analyses.^49^ Industry involvement did however not provide an explanation as a potential source of observed heterogeneity. Moreover, grey literature did not reveal any unpublished studies.

Differences in research models, methodology and outcomes can explain diverse findings. Specifically, the heterogeneous methodology among studies (different period of treatment, study population, percentage of ADS as well type of ADS) may have caused discrepancies among trials.

Nonetheless, the descriptive analysis of this review demonstrates that the majority of the experiments found no differences with respect to bleeding, gingivitis and plaque scores between CHX‐MW and CHX‐MW+ADS. This is confirmed by the meta‐analyses. However, considerable statistical heterogeneity was observed in those meta‐analyses that evaluated the intervention under non‐brushing circumstances. This was not the case for studies that allowed brushing in combination with the mouthrinse intervention.

### 4.6 Statistical heterogeneity

*I* ^2^ is the ratio of true heterogeneity to the total variation in observed effect, which can be interpreted as a signal‐of‐noise ratio. It is not sensitive to the metric of the effect size nor to the number of included studies.^50^ *I* ^2^ was found to be 0%‐26% for brushing studies (see Table 4b). This was interpreted as potentially unimportant with respect to heterogeneity.^22^ For non‐brushing studies in the meta‐analysis, considerable heterogeneity was mainly observed in the end scores and incremental difference scores (*I* ^2^ = 97%‐99%). The observed statistical heterogeneity suggests that the studies were not all estimating the same quantity. On the other hand, it would be surprising if multiple studies, performed by different teams in different places with different methods, all ended up estimating the same underlying parameter.^18^

There are several options to address (statistical) heterogeneity. For the present review, it was chosen to explore heterogeneity by performing sensitivity analysis. This is a repeat of the primary analysis or meta‐analysis, substituting alternative decisions or ranges of values for decisions that were arbitrary or unclear. It is an informal comparison made between different ways of estimating the same thing. Some sensitivity analyses can be prespecified in the study protocol, but often only identified during the review process where the individual peculiarities of the studies under investigation are identified.^18^ The latter was the case during preparation of this systematic review. For the sensitivity analyses, different factors as source of heterogeneity were explored being outliers, study design and sample size. The overall result and conclusions were not affected by the sensitivity analyses although it had an effect on the statistical heterogeneity expressed by *I* ^2^. Consequently, the results of this review can be regarded with a higher degree of certainty. However, when the testing for heterogeneity is significant, the reader should always exercise caution in using the effect size that emerges from the meta‐analysis, because the estimate may not reflect the actual effect in any particular population being studied.^22^

## 5 CONCLUSION

There is moderate quality evidence from non‐brushing studies that the addition of an ADS to CHX‐MW reduces tooth surface discoloration and does not appear to affect its properties with respect to gingival inflammation and plaque scores. In brushing studies, there is also moderate quality evidence that ADS does not affect the anti‐plaque and anti‐gingivitis efficacy of CHX. The majority of comparisons and the meta‐analysis including these indicate no significant effect of ADS on tooth staining in situations where the mouthwash is used in addition to toothbrushing.

## 6 CLINICAL RELEVANCE

### 6.1 Scientific rationale for the study

The most common side effect of chlorhexidine is extrinsic staining of oral surfaces. An anti‐discoloration system presumably reduces staining while maintaining the chlorhexidine efficacy.

### 6.2 Principal findings

A significant benefit was found in favour of chlorhexidine mouthwash with an anti‐discoloration‐system in 4‐21 days non‐brushing studies for stain scores. No differences in the clinical parameters of plaque, bleeding and gingival index scores were found for either brushing or non‐brushing studies (ie experimental gingivitis conditions).

### 6.3 Practical implications

When a chlorhexidine mouthwash is prescribed, there is moderate evidence for 4‐21 days non‐brushing situations, that a product containing an anti‐discoloration system can be considered in order to reduce side effects. This may potentially improve patient compliance.

## CONFLICT OF INTEREST

The authors declare that they have no conflict of interests.

## AUTHOR CONTRIBUTION

All authors gave final approval and agreed to be accountable for all aspects of work ensuring integrity and accuracy. BVS: contributed to design, search and selection, analysis and interpretation, and drafted the manuscript. GAW: contributed to conception and design, analysis and interpretation, and critically revised the manuscript. EB: contributed to analysis and interpretation, and critically revised the manuscript. FG: contributed to design, analysis and interpretation, and critically revised the manuscript. DES: contributed to conception and design, search and selection, analysis and interpretation, and critically revised the manuscript.

## ETHICAL APPROVAL

Ethical approval was not required. van der Weijden, Slot and their research team at ACTA have previously received either external advisor fees, lecturer fees or research grants from dental care product manufacturers. Those manufacturers included GABA/Colgate, Dentaid, Lactona, Oral‐B/Procter & Gamble, Sara Lee, Sunstar Philips Unilever and Waterpik. Graziani have previously received either external advisor fees, lecturer fees or research grants from Curaden, Dentaid, Johnson & Johnson, Oral‐B/Procter & Gamble, Sunstar.
