# Supplementary material for: Does chlorhexidine mouthwash, with an anti‐discoloration system, reduce tooth surface discoloration without losing its efficacy? A systematic review and meta‐analysis
Source: Int J Dent Hyg. 2019 Aug 1;18(1):27–43. doi: 10.1111/idh.12402 (PMC7003798; doi:10.1111/idh.12402)
Supplement: Supplementary file 1 [file IDH-18-27-s001.docx]

**ONLINE Appendix S1.**

Methodological quality and potential risk of bias scores of the individual included studies.

|  | |  |  | | | | |  |  |  |  |  |  |  |  |
| --- | --- | --- | --- | --- | --- | --- | --- | --- | --- | --- | --- | --- | --- | --- | --- |
| **Study**  **Quality criteria** | | **Non-Brushing** | | | | | **Brushing** | | | | | | | | |
|  |  | Arweiler et al. (2006) | Basso et al. (2008) | Li et al.  (2013) | Weinstein & Weinstein. (2014) | Marelli et al. (2015) | Bernardi et al. (2004) | | Graziani et al. (2009) | Solis et al.  (2010) | Amato et al.  (2012) | Graziani et al. (2015) | Pereira et al. (2017) | Varoni et al.  (2017) | Guerra et al.  (2018) |
| **Internal validity** | Study design | cross over | parallel | parallel | cross  over | parallel | cross  over | | parallel | cross  over | parallel | parallel | cross over | cross over | parallel |
|  | Random allocation* | *+* | NR | + | + | + | + | | NR | + | + | + | + | + | + |
|  | Allocation concealment | NR | NR | NR | NR | NR | NR | | NR | NR | + | + | NR | + | + |
|  | Blinded to product* | + | + | + | + | + | + | | + | + | + | + | + | + | + |
|  | Blinded to examiner* | + | + | + | *+* | + | *NR* | | *+* | *+* | + | + | + | + | + |
|  | Blinding during statistical analysis | NR | NR | NR | NR | NR | NR | | NR | NR | + | NR | NR | *+* | NR |
|  | Balanced experimental groups* | *+* | *+* | *+* | *+* | + | *+* | | + | + | + | + | + | + | + |
|  | Reported loss to follow-up* | + | + | + | + | + | + | | + | + | + | + | + | + | + |
|  | # (%)  of drop-outs | 2(9.5%) | 0 (0%) | 0 (0%) | 0 (0%) | 0 (0%) | 0 (0%) | | 0 (0%) | 2 (11.8%) | 0 (0%) | 0 (0%) | 0 (0%) | 0(0%) | 2 (3%) |
|  | Treatment identical, except for intervention* | + | + | + | - | + | + | | + | + | + | + | + | + | + |
| **External validity** | Representative population group | ± | + | ± | + | + | + | | + | + | + | + | ± | ± | + |
|  | Eligibility criteria defined* | + | + | + | + | + | + | | + | + | + | + | + | + | + |
|  | Sample size calculation and power | + | - | + | + | - | - | | - | - | - | + | - | + | + |
|  | Point estimates presented for the primary outcome mean | + | - | + | + | + | + | | + | + | + | + | + | + | + |
|  | Measures of variability presented for the primary outcome SD/ci | + | - | + | - | - | + | | + | + | + | + | + | + | + |
|  | Unit of analysis | Subject | Subject | Subject | Subject | Subject | Subject | | Subject | Subject | Subject | Subject | Subject | Subject | Subject |
|  | Included a per protocol analysis | NR | + | + | + | + | + | | *+* | *+* | + | + | + | - | + |
|  | Included an intention-to-treat analysis | NR | - | - | - | - | - | | *-* | *-* | - | - | - | + | - |
| **Clinical aspects** | Validated measurement | + | - | + | + | + | + | | + | + | + | + | - | + | + |
|  | Calibration examiner | + | NR | + | NR | - | NR | | NR | NR | NR | + | NR | NR | NR |
|  | Reproducibility data shown | + | - | + | + | - | + | | + | + | + | + | + | - | + |
| Authors’ estimated risk of bias | | **Low** | **Moderate** | **Low** | **Low** | **Low** | **Moderate** | | **Low** | **Low** | **Low** | **Low** | **Low** | **Low** | **Low** |

Each aspect of the score list was given a rating of ‘+’ for an informative description of the item at hand and a study design meeting the quality standard,

‘-’ for an informative description without a study design that met the quality standard and ‘?’ for missing or insufficient information. When random allocation, defined eligibility criteria, blinding of examiners and patients, balanced experimental groups, identical treatment between groups (except for intervention) and report of follow-up were present, the study was classified as having a low risk of bias. When one of these seven criteria was missing, the study was considered to have a moderate potential risk of bias. When two or more of these criteria were missing, the study was considered to have a high potential risk of bias, as proposed by Van der Weijden et al. (2009)^21^.

* reporting criteria for estimating the potential risk of bias

NR: not reported

**ONLINE Appendix S2.**

Mean (SD) scores for the different intervention groups with various indices and their modifications. Within-group analyses are presented. S2a: Staining index (SI), S2b: Plaque Index (PI), S2c: Bleeding Index(BI), S2d: Gingival Index (GI).

| **# SI** | **Index %** | **Group** | **Baseline** | **End** | **Difference** |
| --- | --- | --- | --- | --- | --- |
| **NON-BRUSHING** | | | | | |
| Basso et al.  (2008) | Lobene  (1963)  Extent (area)  CIE-Lab | CHX-MW + ADS | 0 (0)♦ | 0.77 (0.76)♦ | 0.77 (0.76)♦ |
|  |  | CHX-MW | 0 (0)♦ | 1.43 (1.02)♦ | 1.43 (1.02)♦ |
| Marelli et al.  (2015) | Lobene  (1968) Intensity + extent (area) | CHX-MW + ADS (D) | 0.56 (0.03♦) | 0.49(0.01♦) | -0.07(0.031♦) |
|  |  | CHX-MW (C) | 0.68(0.11♦) | 0.81(0.09♦) | 0.13(0.01♦) |
|  |  | CHX-MW (E) | 0.59(0.07♦) | 0.64(0.03♦) | 0.05(0.12♦) |
| Weinstein & Weinstein  (2014) | Lobene  (1986) | CHX-MW + ADS | 0.057(0.070)♦ | 0.498(0.203)♦ | 0.441(0.182)♦ |
|  |  | CHX-MW | 0.049(0.062)♦ | 0.857(0.297)♦ | 0.808(0.306)♦ |
| Li et al. *  (2013) | Lang & Raber (1981) Intensity | CHX-MW + ADS (T2) | 0.03(0.04) | 0.21(0.17) | 0.18(0.15♦) |
|  |  | CHX-MW (T1) | 0.02(0.03) | 1.13(0.59) | 1.11(0.56♦) |
| **BRUSHING** | | | | | |
| Graziani et al.  (2015) | Lobene  (1968)  CIE-Lab  Extent (area) | CHX-MW + ADS (CHX3) | - | 22.6 (27.2)♦ |  |
|  |  | CHX-MW alc- (CHX2) | - | 17.8(25.2)♦ |  |
|  |  | CHX-MW alc+ (CHX1) | - | 29.3 (30.1)♦ |  |
| Bernardi et al.  (2004) | CIELAB system | CHX-MW + ADS (B) | (?) | (?) | (?) |
|  |  | CHX-MW (A) | (?) | (?) | (?) |
| Varoni et al.  (2017) | CIELAB system | CHX-MW + ADS |  | 8.622(6.377)♦ |  |
|  |  | CHX-MW |  | 8.486(5.541)♦ |  |
| Solis et al.  (2010) | Brecx (1993) | CHX-MW + ADS (A) | 0.205(0.194) | 0.521(0.337) | 0.316(?) |
|  |  | CHX-MW (B) | 0.441(0.344) | 0.953(0.484) | 0.512(?) |
| Pereira et al.  (2017) | Li  (2014) Intensity | CHX-MW + ADS | ‡ | 0.4118(0.0399)♦ | 0.4118(0.0399)♦ |
|  |  | CHX-MW | ‡ | 0.5668(0.04114)♦ | 0.5668(0.04114)♦ |
| Guerra et al.  (2018) Manuscript | CIELAB system | CHX-MW + ADS | (?) | (?) | (?) |
|  |  | CHX-MW | (?) | (?) | (?) |
| Amato et al (2011) |  | CHX-MW + ADS | (?) | 11.38 | (?) |
|  |  | CHX-MW | (?) | 38.55 | (?) |

| **# PI** | **Index %** | **Group** | **Baseline** | **End** | **Difference** |
| --- | --- | --- | --- | --- | --- |
| **NON-BRUSHING** | | | | | |
| Arweiler et al.  (2006) | Silness and Loe  (1964) | CHX-MW + ADS | 0.67(0.33) | 1.00(0.44) | 0.33(0.34) ♦ |
|  |  | CHX-MW | 0.42(0.21) | 0.43(0.33) | 0.01(0.35) ♦ |
| Basso et al.  (2008) | Silness and Löe  (1964) | CHX-MW + ADS | 0(0)♦ | 1.45 (0.76)♦ | 1.45 (0.76)♦ |
|  |  | CHX-MW | 0(0)♦ | 1.38 (0.8)♦ | 1.38 (0.8)♦ |
| Li et al.  (2013) | Silness and Löe  (1964) | CHX-MW + ADS (T2) | 0.06(0.06) | 1.45(0.39) | 1.39 (0.46) ♦ |
|  |  | CHX-MW (T1) | 0.06(0.05) | 0.25(0.19) | 0.19 (0.12) ♦ |
| Weinstein & Weinstein  (2014) | Silness and Löe  (1963) | CHX-MW + ADS | 0.116(0.088)♦ | 0.351(0.193)♦ | 0.236(0.169)♦ |
|  |  | CHX-MW | 0.105(0.108)♦ | 0.308(0.172)♦ | 0.203(0.170)♦ |
| Marelli et al.  (2015) | Silness and Löe  (1963) | CHX-MW + ADS (D) | 1.82 (0.27♦) | 1.44 (0.01♦) | -0.38(0.15♦) |
|  |  | CHX-MW (C) | 1.88 (0.31♦) | 1.85 (0.02♦) | -0.03 (0.074♦) |
|  |  | CHX-MW (E) | 2.0 (0.77♦) | 2.04 (0.21♦) | 0.04(0.02♦) |
| **BRUSHING** | | | | | |
| Bernardi et al.  (2004) | Silness and Löe  (…) | CHX-MW + ADS (B) | 1(0.17) | 0.2(0.11) | 0.8(?) |
|  |  | CHX-MW (A) | 1(0.17) | 0.2(0.11) | 0.8(?) |
| Solis et al.  (2010) | Silness and Löe  (1964) | CHX-MW + ADS (A) | ‡ | 0.087(0.129) | NA |
|  |  | CHX-MW (B) | ‡ | 0.175(0.267) | NA |
| Pereira et al.  (2017) | Löe (1967) | CHX-MW + ADS | ‡ | 0.2904(0.0347)♦ | NA |
|  |  | CHX-MW | ‡ | 0.2678(0.0252)♦ | NA |
| Varoni et al.  (2017) | Sillness & Loe (1967) | CHX-MW + ADS | 0.041(0.084)♦ | 0.0092(0.019)♦ | 0.032(0.087)♦ |
|  |  | CHX-MW | 0.046(0.068)♦ | 0.006(0.016)♦ | 0.039(0.073)♦ |
| Graziani et al.  (2015) | O’Leary  (1972) | CHX-MW alc- + ADS (CHX3) | 41.4 (20.5)◊ | 17.8 (8.6)◊ | 23.6(?) |
|  |  | CHX-MW alc- (CHX2) | 40.0 (28.9)◊ | 10.1 (7.1)◊ | 29.9(?) |
|  |  | CHX-MW alc+ (CHX1) | 44.3 (29.3)◊ | 11.5 (8.8)◊ | 32.8(?) |
| Guerra et al.  (2018) | O’Leary  (1972) | CHX-MW + ADS | 62.67(16.87) | (?) | 30.67(15.22) |
|  |  | CHX-MW | 55.17(14.31) | (?) | 19.93(11.03) |
| Amato et al (2011) | FMPS | CHX-MW + ADS | 51.7(6.5) | 17.7(2.0) | (?) |
|  |  | CHX-MW | 50.6(5.4) | 20.2(2.4) | (?) |

| **# GI** | **Index %** | **Group** | **Baseline** | **End** | **Difference** |
| --- | --- | --- | --- | --- | --- |
| **NON-BRUSHING** | | | | | |
| Basso et al.  (2008) | Löe and Silness  (1963) | CHX-MW + ADS | 0(0)♦ | 0.15 (0.34)♦ | 0.15 (0.34)♦ |
|  |  | CHX-MW | 0(0)♦ | 0.17 (0.37)♦ | 0.17 (0.37)♦ |
| Li et al.  (2013) | Löe and Silness  (1963) | CHX-MW + ADS (T2) | 0.05(0.04) | 1.12(0.41) | 1.07 (0.38♦) |
|  |  | CHX-MW (T1) | 0.06(0.05) | 0.18(0.19) | 0.12 (0.10♦) |
| Weinstein & Weinstein  (2014) | Löe  (1967) | CHX-MW + ADS | 0.137(0.118)♦ | 0.347(0.179)♦ | 0.210(0.164)♦ |
|  |  | CHX-MW | 0.143(0.115)♦ | 0.334(0.160)♦ | 0.191(0.143)♦ |
| **BRUSHING** | | | | | |
| Solis et al.  (2010) | Löe  (1967) | CHX-MW + ADS (A) | ‡ | 0.082(0.085) | NA |
|  |  | CHX-MW (B) | ‡ | 0.072(0.072) | NA |
| Graziani et al.  (2015) | Löe  (1967) | CHX-MW alc- + ADS (CHX 3) | 1.8 (0.7♦) | 0.6 (0.5♦) | 1.2 (?) |
|  |  | CHX-MW alc- (CHX2) | 1.6 (0.7♦) | 0.6 (0.6♦) | 1.0 (?) |
|  |  | CHX-MW alc+ (CHX1) | 1.8 (0.7♦) | 0.9 (0.6♦) | 0.9 (?) |
| Bernardi et al.  (2004) | Silness and Löe  (?) | CHX-MW + ADS (B) | 0.53(0.13) | 0 | -0.53(?)◊ |
|  |  | CHX-MW (A) | 0.53(0.13) | 0 | -0.53(?)◊ |

| **# BI** | **Index %** | **Group** | **Baseline** | **End** | **Difference** |
| --- | --- | --- | --- | --- | --- |
| **BRUSHING** | | | | | |
| Graziani et al.  (2015) | Ainamo and Bay  (1975) | CHX-MW alc- + ADS (CHX3) | 16.9(16.2)◊ | (?) | (?) |
|  |  | CHX-MW alc- (CHX2) | 15.1 (18.3)◊ | (?) | (?) |
|  |  | CHX-MW alc+ (CHX 1) | 19.7(24.4)◊ | (?) | (?) |
| Varoni et al.  (2017) | Caton and Polson (1985) | CHX-MW + ADS |  |  |  |
|  |  | CHX-MW |  |  |  |
| Guerra et al.  (2018) Manuscript | Ainamo and Bay  (1975) | CHX-MW + ADS | 21.50(12.17) | (?) | -9.82(9.27) |
|  |  | CHX-MW | 30.93(20.75) | (?) | -19.31(11.33) |
| Amato et al (2011) | FMBS | CHX-MW + ADS | 50.0(5.8) | 18.1(3.0) | (?) |
|  |  | CHX-MW | 48.8(5.6) | 17.8(2.6) | (?) |

SI: Staining Index, PI: Plaque Index, BI: Bleeding Index, GI: Gingival Index

CHX: Chlorhexidine, ADS, Anti-discoloration System, MW: Mouthwash,

Alc+: With Alcohol, Alc-: Without Alcohol, Alc?: Not Reported With or Without Alcohol

? Unknown/not-provided

₪ Insufficient data presentation

◊ Calculated by the authors of this review based on the presented data in the selected paper

♦ Provided by the original author

‡ Not measured
NA Not applicable

**ONLINE Appendix S3**

Forrest plots using a random model of the performed meta-analysis on the efficacy of CHX-MW+ADS on the staining scores analysed with the Standardized Mean Difference (SMD) for non-brushing studies.

1. Baseline


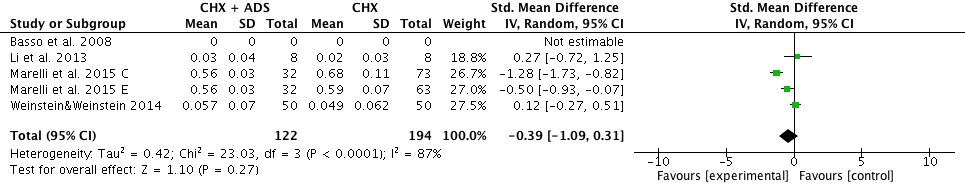


1. End


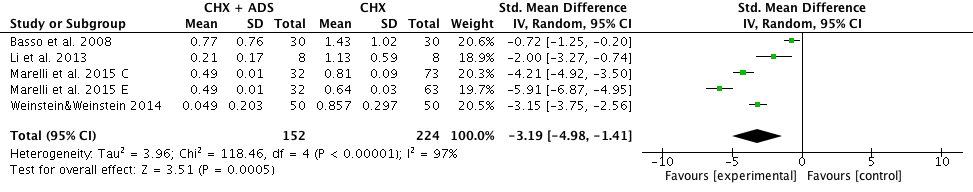


1. Difference

**
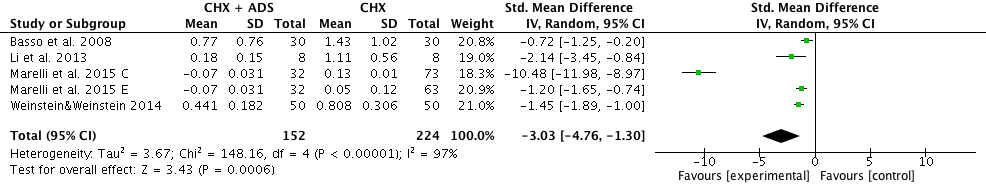
**

**ONLINE Appendix S4**

Forrest plots using a random model of the performed meta-analysis on the efficacy of CHX-MW+ADS on the plaque scores analysing Silness and Löe (1964)^39^ for non-brushing studies.

1. Baseline


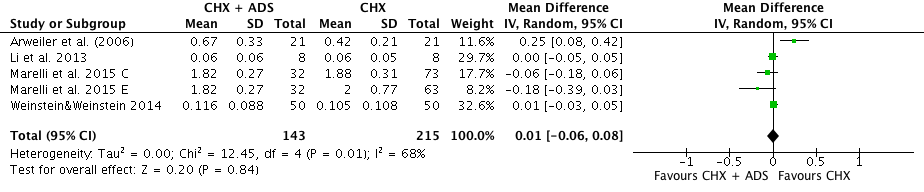


1. End


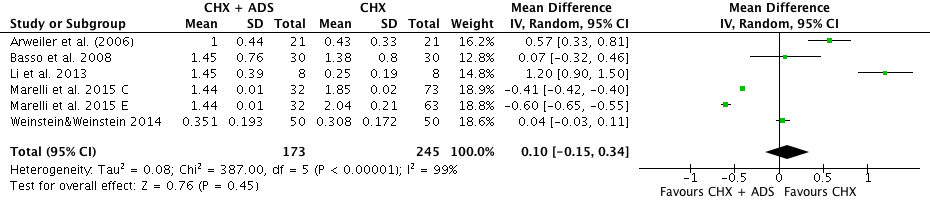


1. Difference


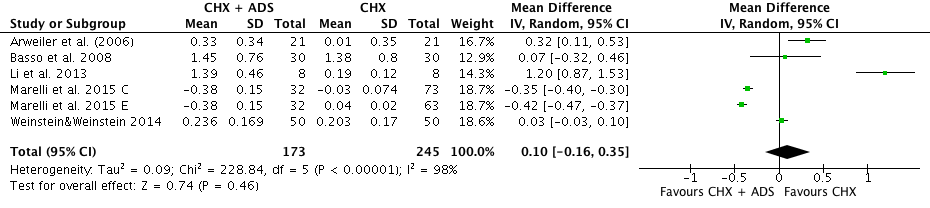


**ONLINE Appendix S5**

Forrest plots using a fixed model of the performed meta-analysis on the efficacy of CHX-MW+ADS on the gingival index scores analysing Löe and Silness (1963)^40^ for non-brushing studies.

1. Baseline


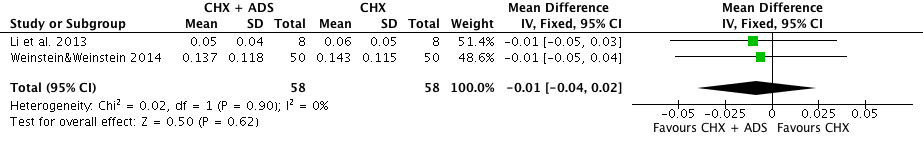


1. End


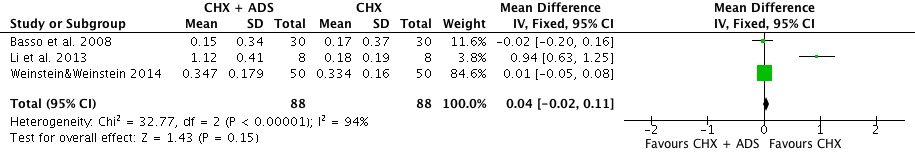


1. Difference


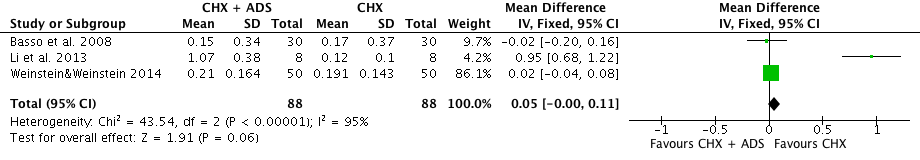


**ONLINE Appendix S6**

Forrest plots using a fixed model of the performed meta-analysis on the efficacy of CHX-MW+ADS on the staining scores analysed with the Standardized Mean Difference (MD) for brushing studies.

1. End


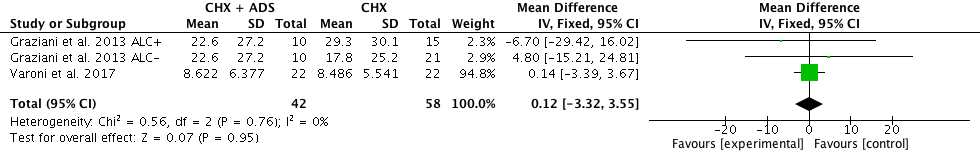


**ONLINE Appendix S7**

Forrest plots using a random model of the performed meta-analysis on the efficacy of CHX-MW+ADS on the plaque scores analysing Silness and Löe (1964)^39^ for brushing studies.

1. End
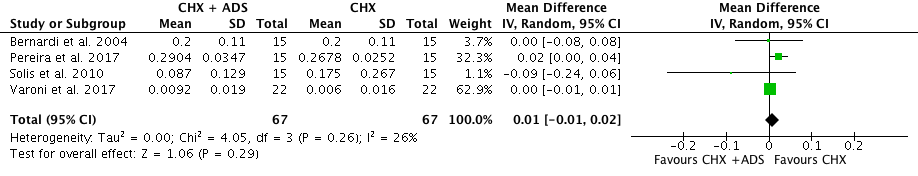


**ONLINE Appendix S8**

Forrest plots using a fixed model of the performed meta-analysis on the efficacy of CHX-MW+ADS on the gingival index scores analysing Löe and Silness (1963)^40^ for brushing studies.

1. Baseline

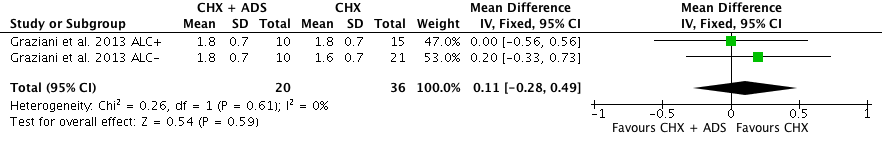

2. End

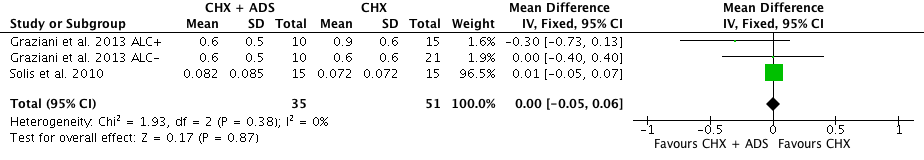


**ONLINE Appendix S9**

Sub analysis: Forrest plots of the performed meta-analysis on the efficacy of CHX-MW+ADS for low risk of bias studies.

1. Gingival index, non-brushing (end)


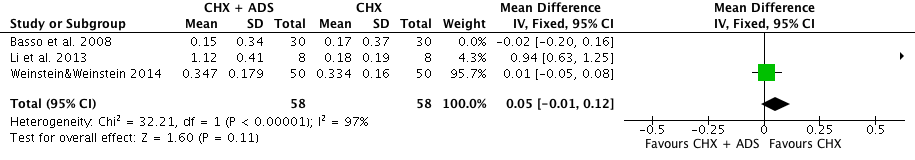


1. Plaque index, non-brushing (end)


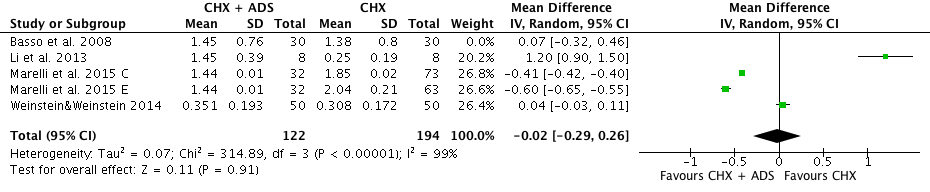


1. Plaque index, brushing (end)


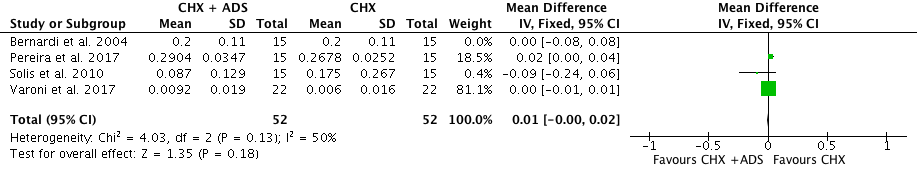


**ONLINE Appendix S10**

List of abbreviations

| **Abbreviation** | **Meaning** |
| --- | --- |
| ADS | Anti-discoloration-system |
| Alc- | Without alcohol |
| Alc+ | With alcohol |
| Alc? | Not Reported With or Without Alcohol |
| B | Brushing |
| BI | Bleeding Index |
| BVS | Bregje van Swaaij; first author of this paper |
| CCT | Controlled Clinical Trial |
| CHX | Chlorhexidine |
| DES | Dagmar Else Slot; co-author of this paper |
| GAW | Godefridus August van der Weijden; co-author of this paper |
| GI | Gingival Index |
| IDC | Interdental Cleaning |
| SD | Standard Deviation |
| SR | Systematic Review |
| MA | Meta-analysis |
| MW | Mouthwash |
| NA | Not Applicable |
| NB | Non Brushing |
| N.R. | Not Reported |
| OP | Oral prophylaxis |
| PI | Plaque Index |
| PVP-VA | Polyvinylpyrrolidon Vinylacetat |
| RCT | Randomized Controlled Trial |
| RCCT | Randomized Clinical Controlled Trial |
| SI | Staining Index |
